# Supplementary material for: Green Manuring Enhances Soil Multifunctionality in Tobacco Field in Southwest China
Source: Microorganisms. 2024 May 7;12(5):949. doi: 10.3390/microorganisms12050949 (PMC11124463; doi:10.3390/microorganisms12050949)
Supplement: Supplementary file 1 [file microorganisms-12-00949-s001.zip › microorganisms-2960435-supplementary.pdf]

Supplementary Material for

This file includes:

1. Supplementary Figures S1 to S6

2. Supplementary Tables S1 to S5

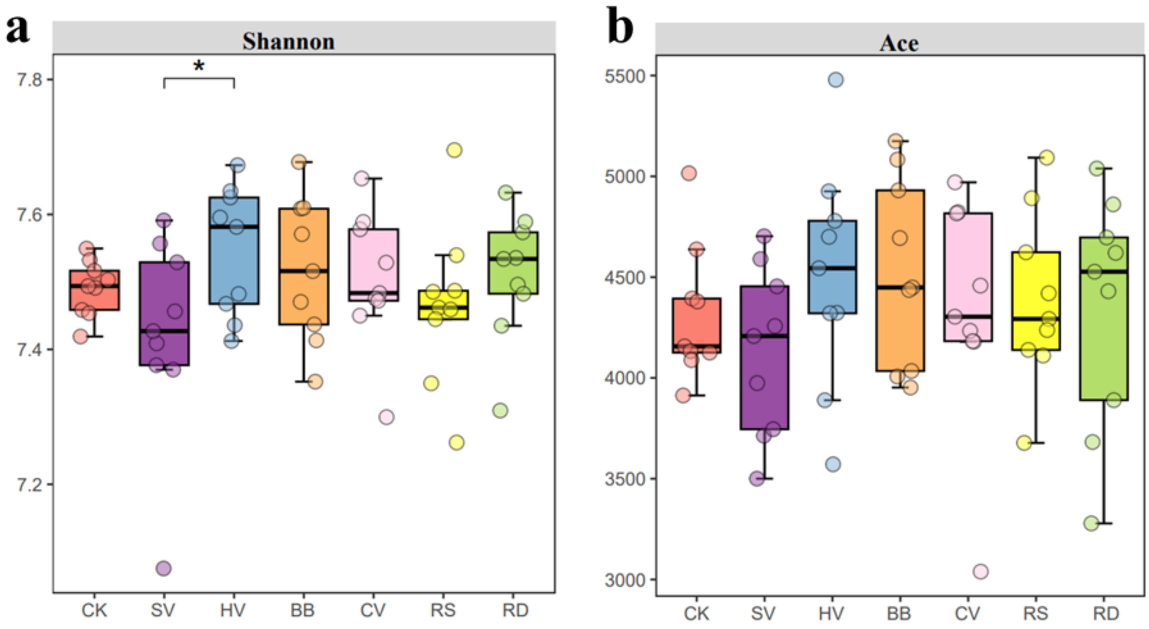

Figure S1. Shannon (a) index and ACE (b) index analysis of bacterial communities in rhizosphere soil. CK—winter fallow control; SV—returning smooth vetch; HV—returning hairy vetch; BB—returning broad bean; CV—returning common vetch; RS—returning rapeseed; RD—returning radish. Different lowercase letters at the tops of boxes indicate significant differences among treatments at the same stage (P < 0.05).

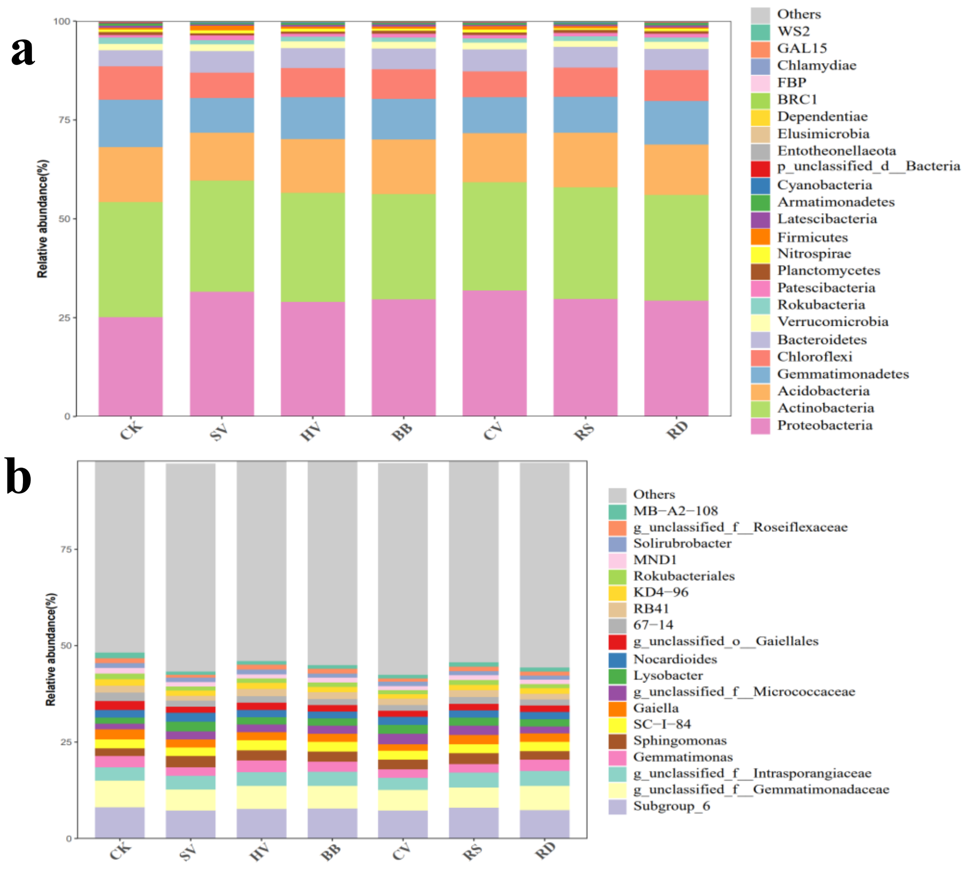

Figure S2 Composition of soil bacterial community at phylum (a) and genus (b) level. CK—winter fallow control; SV—returning smooth vetch; HV—returning hairy vetch; BB—returning broad bean; CV—returning common vetch; RS—returning rapeseed; RD—returning radish. Different lowercase letters at the tops of boxes indicate significant differences among treatments at the same stage (P < 0.05).

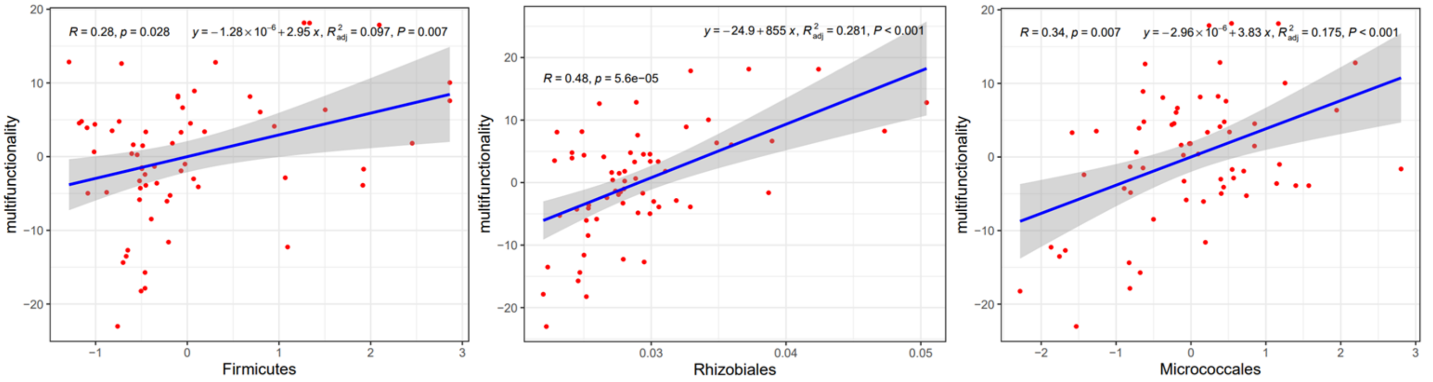

Figure S3 Regression analysis of significantly different species in bacteria and soil multifunctionality.

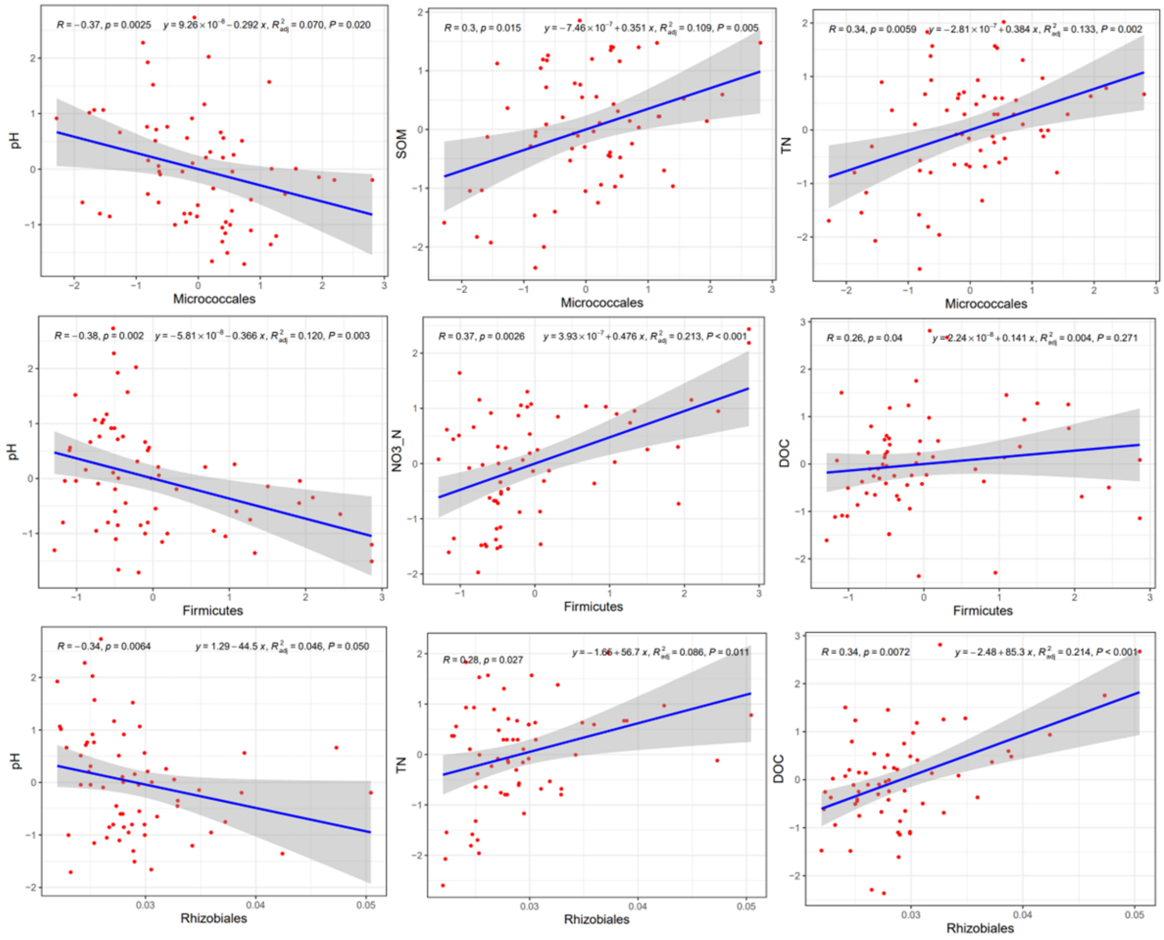

Figure S4 Regression analysis of significantly different species in bacteria and environmental factors.

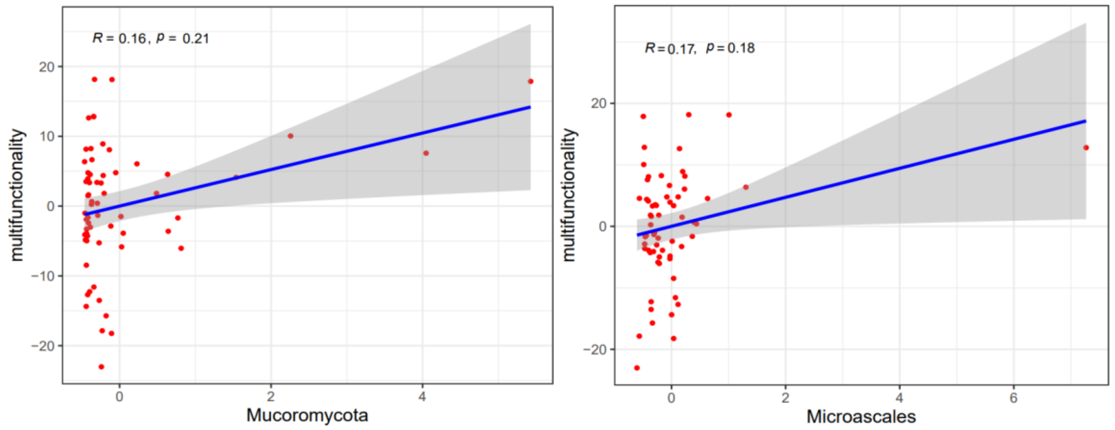

Figure S5 Regression analysis of different fungal species and soil multifunctionality in SV and CV treatments

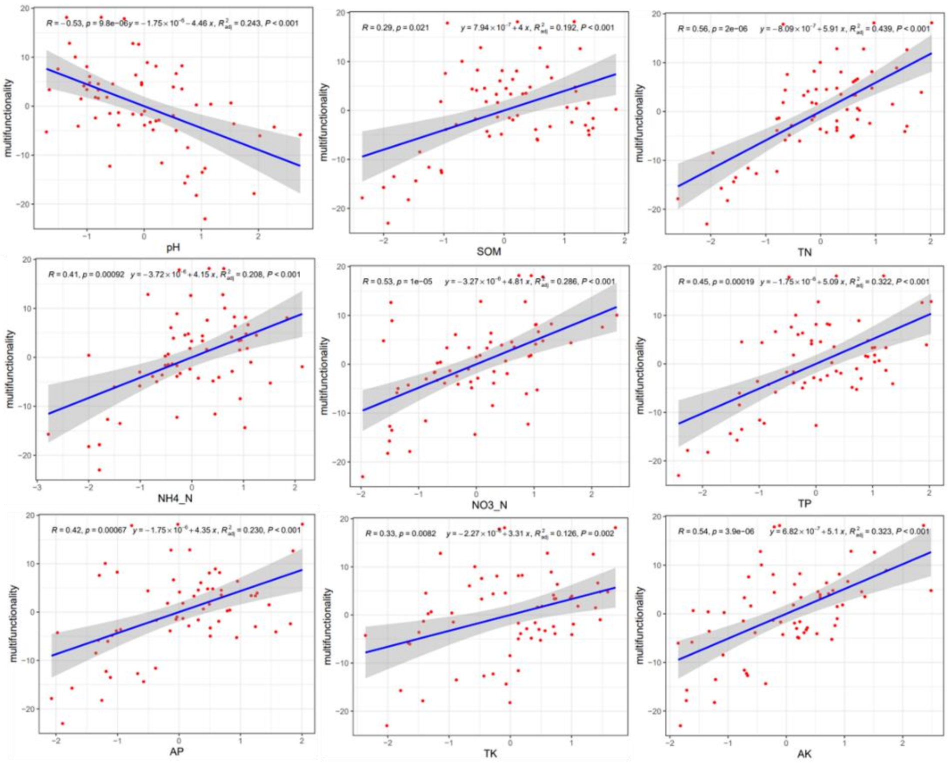

Figure S6 Regression analysis of environmental factors and soil multifunctionality

Table S1 Two-way ANOVA of sampling date, green manure treatment, and their interaction on rhizosphere soil microbial diversity.

	Dependent variable	Df	Bacterial		Fungal	
			F	P	F	P
Sampling date (S)	Shannon	2	0.536	0.589	1.045	0.36
	ACE	2	1.679	0.199	7.159	0.002**
Treatment (T)	Shannon	6	1.615	0.167	1.195	0.328
	ACE	6	0.855	0.536	7.092	0.001***
S * T	Shannon	12	2.315	0.022*	1.152	0.348
	ACE	12	2.216	0.029*	0.784	0.664

Bacterial: $R^2_{shannon}$=0.479 R^2_{ACE}=0.455. Fungal: $R^2_{shannon}$=0.355 R^2_{ACE}=0.612

* P < 0.05 ** P < 0.01; *** P < 0.001

Table S2. Two-way ANOVA of sampling date, green manure treatment, and their interaction on flue-cured tobacco soil multifunctionality

	Dependent variable	Df	F	P
Sampling date (S)		2	2.293	0.113

Treatment (T)	Soil multifunctionality	6	23.905	0.001***
S* T		12	3.577	0.001**

R^2 =0.820; ** $P < 0.01$; *** $P < 0.001$

Table S3 Taxonomic composition of key ecological modules

ASV	Abundance	Type	Genus	Phylum	Module
**fungi_ASV_4550**	0.03983	fungi	Fusarium	Ascomycota	2
**fungi_ASV_5732**	0.029781	fungi	Plectosphaerella	Ascomycota	2
fungi_ASV_3339	0.015299	fungi	Penicillium	Ascomycota	2
bac_ASV_95240	0.005666	bac	Sphingobium	Proteobacteria	2
fungi_ASV_899	0.003349	fungi	Fusarium	Ascomycota	2
fungi_ASV_8750	0.002799	fungi	Staphylotrichum	Ascomycota	2
bac_ASV_84909	0.002676	bac	g_unclassified_f__Intrasporangiaceae	Actinobacteria	2
bac_ASV_109816	0.002212	bac	g_unclassified_o__Azospirillales	Proteobacteria	2
fungi_ASV_1327	0.002065	fungi	g_unclassified_o__Eurotiales	Ascomycota	2
bac_ASV_60856	0.001969	bac	Rokubacteriales	Rokubacteria	2
bac_ASV_61744	0.0019	bac	Phycicoccus	Actinobacteria	2
bac_ASV_65416	0.001797	bac	g_unclassified_f__Intrasporangiaceae	Actinobacteria	2
bac_ASV_81152	0.001716	bac	g_unclassified_f__Gemmatimonadaceae	Gemmatimonadetes	2
bac_ASV_37166	0.001667	bac	g_unclassified_f__Gemmatimonadaceae	Gemmatimonadetes	2
bac_ASV_43339	0.001549	bac	Subgroup_6	Acidobacteria	2
bac_ASV_31257	0.001533	bac	RB41	Acidobacteria	2
bac_ASV_14847	0.001449	bac	Rhodanobacter	Proteobacteria	2
bac_ASV_110881	0.001439	bac	Subgroup_6	Acidobacteria	2
bac_ASV_90271	0.001435	bac	bacteriap25	Proteobacteria	2
bac_ASV_26988	0.001349	bac	g_unclassified_f__Gemmatimonadaceae	Gemmatimonadetes	2
bac_ASV_99421	0.001311	bac	MB_A2_108	Actinobacteria	2
bac_ASV_43507	0.001306	bac	g_unclassified_f__Gemmatimonadaceae	Gemmatimonadetes	2
bac_ASV_110025	0.001232	bac	g_unclassified_f__Gemmatimonadaceae	Gemmatimonadetes	2
**fungi_ASV_319**	0.001077	fungi	Verticillium	Ascomycota	2
bac_ASV_34763	0.001068	bac	g_unclassified_f__Gemmatimonadaceae	Gemmatimonadetes	2
bac_ASV_43917	0.001067	bac	mle1_7	Proteobacteria	2
bac_ASV_43987	0.001067	bac	Sphingobium	Proteobacteria	2
bac_ASV_70065	0.001025	bac	g_unclassified_f__Gemmatimonadaceae	Gemmatimonadetes	2
bac_ASV_65171	0.000997	bac	TK10	Chloroflexi	2
bac_ASV_83960	0.000983	bac	MB_A2_108	Actinobacteria	2
bac_ASV_68900	0.000973	bac	Opitutus	Verrucomicrobia	2
bac_ASV_50022	0.000945	bac	Subgroup_6	Acidobacteria	2
bac_ASV_77978	0.000926	bac	g_unclassified_o__Microtrichales	Actinobacteria	2
bac_ASV_141	0.0009	bac	g_unclassified_f__Ilumatobacteraceae	Actinobacteria	2
bac_ASV_86790	0.00089	bac	Ramlibacter	Proteobacteria	2
bac_ASV_82069	0.000884	bac	Gemmatimonas	Gemmatimonadetes	2
bac_ASV_19526	0.000868	bac	S0134_terrestrial_group	Gemmatimonadetes	2
bac_ASV_82654	0.000831	bac	Blastococcus	Actinobacteria	2
bac_ASV_12046	0.0008	bac	Geodermatophilus	Actinobacteria	2
bac_ASV_18157	0.000798	bac	Subgroup_6	Acidobacteria	2
bac_ASV_26325	0.00078	bac	Agromyces	Actinobacteria	2
bac_ASV_43990	0.000731	bac	MND1	Proteobacteria	2
bac_ASV_17299	0.000724	bac	g_unclassified_f__Microscillaceae	Bacteroidetes	2
bac_ASV_28798	0.000724	bac	67_14	Actinobacteria	2
bac_ASV_44018	0.00072	bac	Subgroup_6	Acidobacteria	2
fungi_ASV_6687	0.000713	fungi	Penicillium	Ascomycota	2
bac_ASV_104420	0.000702	bac	g_unclassified_f__Gemmatimonadaceae	Gemmatimonadetes	2

bac_ASV_116079	0.000702	bac	Subgroup_7	Acidobacteria	2
fungi_ASV_6709	0.000687	fungi	g_unclassified_d__Fungi	p_unclassified_d__Fungi	2
bac_ASV_64161	0.00068	bac	Flavisolibacter	Bacteroidetes	2
bac_ASV_34930	0.000672	bac	Agromyces	Actinobacteria	2
bac_ASV_116355	0.000662	bac	Methylotenera	Proteobacteria	2
bac_ASV_104996	0.000622	bac	g_unclassified_f__Ilumatobacteraceae	Actinobacteria	2
bac_ASV_67415	0.000621	bac	Solirubrobacter	Actinobacteria	2
bac_ASV_108883	0.00062	bac	Blastococcus	Actinobacteria	2
bac_ASV_34024	0.000614	bac	Subgroup_6	Acidobacteria	2
bac_ASV_22569	0.000603	bac	AKAU4049	Gemmatimonadetes	2
bac_ASV_20311	0.000598	bac	Subgroup_22	Acidobacteria	2
bac_ASV_75100	0.000593	bac	g_unclassified_f__Gemmatimonadaceae	Gemmatimonadetes	2
bac_ASV_38457	0.000588	bac	g_unclassified_f__Intrasporangiaceae	Actinobacteria	2
bac_ASV_31557	0.000558	bac	Gemmatimonas	Gemmatimonadetes	2
bac_ASV_112002	0.000542	bac	Ilumatobacter	Actinobacteria	2
bac_ASV_37940	0.000529	bac	g_unclassified_f__Gemmatimonadaceae	Gemmatimonadetes	2
bac_ASV_26189	0.000519	bac	RB41	Acidobacteria	2
bac_ASV_63116	0.000502	bac	Subgroup_10	Acidobacteria	2
bac_ASV_62040	0.000489	bac	g_unclassified_f__Gemmatimonadaceae	Gemmatimonadetes	2
bac_ASV_87569	0.000478	bac	Gemmatimonas	Gemmatimonadetes	2
bac_ASV_68226	0.000475	bac	g_unclassified_f__Roseiflexaceae	Chloroflexi	2
bac_ASV_54260	0.000459	bac	Hyphomicrobium	Proteobacteria	2
bac_ASV_2037	0.000458	bac	S0134_terrestrial_group	Gemmatimonadetes	2
bac_ASV_65870	0.000419	bac	Aridibacter	Acidobacteria	2
bac_ASV_110069	0.000418	bac	Bryobacter	Acidobacteria	2
bac_ASV_78281	0.000411	bac	Subgroup_6	Acidobacteria	2
bac_ASV_24463	0.00041	bac	Gemmatirosa	Gemmatimonadetes	2
bac_ASV_41785	0.000408	bac	g_unclassified_c__Alphaproteobacteria	Proteobacteria	2
bac_ASV_94061	0.000408	bac	OM190	Planctomycetes	2
bac_ASV_76398	0.000407	bac	Gitt_GS_136	Chloroflexi	2
bac_ASV_52454	0.0004	bac	C0119	Chloroflexi	2
bac_ASV_71511	0.000386	bac	g_unclassified_f__Xanthobacteraceae	Proteobacteria	2
bac_ASV_96730	0.000386	bac	g_unclassified_f__Intrasporangiaceae	Actinobacteria	2
bac_ASV_4049	0.000377	bac	NB1_j	Proteobacteria	2
bac_ASV_68686	0.000372	bac	Ramlibacter	Proteobacteria	2
bac_ASV_80614	0.000368	bac	Sphingobium	Proteobacteria	2
bac_ASV_25966	0.000366	bac	Chthonomonadales	Armatimonadetes	2
bac_ASV_65554	0.000353	bac	Gemmatimonas	Gemmatimonadetes	2
bac_ASV_33058	0.00035	bac	g_unclassified_f__Gemmatimonadaceae	Gemmatimonadetes	2
bac_ASV_58043	0.000349	bac	g_unclassified_f__Opitutaceae	Verrucomicrobia	2
bac_ASV_6581	0.000347	bac	Gaiella	Actinobacteria	2
bac_ASV_7573	0.000347	bac	g_unclassified_f__Intrasporangiaceae	Actinobacteria	2
bac_ASV_59574	0.000346	bac	Subgroup_6	Acidobacteria	2
bac_ASV_38200	0.000345	bac	Subgroup_6	Acidobacteria	2
bac_ASV_93850	0.00034	bac	Subgroup_6	Acidobacteria	2
fungi_ASV_1252	0.00033	fungi	g_unclassified_d__Fungi	p_unclassified_d__Fungi	2
bac_ASV_38000	0.000317	bac	JG30_KF_CM45	Chloroflexi	2
bac_ASV_42866	0.000314	bac	g_unclassified_f__Gemmatimonadaceae	Gemmatimonadetes	2
bac_ASV_113495	0.000301	bac	Stenotrophobacter	Acidobacteria	2

bac_ASV_67391	0.000294	bac	67_14	Actinobacteria	2
bac_ASV_77010	0.00028	bac	Rokubacteriales	Rokubacteria	2
bac_ASV_72653	0.000277	bac	g_unclassified_o__Gaiellales	Actinobacteria	2
bac_ASV_43040	0.000251	bac	g_unclassified_f__Intrasporangiaceae	Actinobacteria	2
bac_ASV_2969	0.000247	bac	NB1_j	Proteobacteria	2
bac_ASV_98127	0.000237	bac	C0119	Chloroflexi	2
bac_ASV_70960	0.000235	bac	RB41	Acidobacteria	2
bac_ASV_83395	0.000228	bac	NB1_j	Proteobacteria	2
bac_ASV_60610	0.000185	bac	g_unclassified_c__Alphaproteobacteria	Proteobacteria	2
bac_ASV_16973	0.000184	bac	AKYG587	Planctomycetes	2
bac_ASV_1442	0.000172	bac	g_unclassified_f__Gemmatimonadaceae	Gemmatimonadetes	2
bac_ASV_62792	0.000124	bac	g_unclassified_f__Chitinophagaceae	Bacteroidetes	2
bac_ASV_83953	0.000122	bac	Gaiella	Actinobacteria	2
fungi_ASV_2220	0.012798	fungi	Talaromyces	Ascomycota	3
bac_ASV_116505	0.006283	bac	g_unclassified_f__Intrasporangiaceae	Actinobacteria	3
**fungi_ASV_2412**	0.005528	fungi	Chaetomium	Ascomycota	3
bac_ASV_75831	0.00442	bac	Gaiella	Actinobacteria	3
bac_ASV_18811	0.003859	bac	g_unclassified_f__Sporichthyaceae	Actinobacteria	3
bac_ASV_55972	0.00354	bac	MND1	Proteobacteria	3
**fungi_ASV_4781**	0.003444	fungi	g_unclassified_o__Sordariales	Ascomycota	3
fungi_ASV_6061	0.00233	fungi	g_unclassified_f__Sporormiaceae	Ascomycota	3
fungi_ASV_5260	0.002217	fungi	g_unclassified_f__Nectriaceae	Ascomycota	3
fungi_ASV_9401	0.002197	fungi	g_unclassified_o__Hypocreales	Ascomycota	3
bac_ASV_107208	0.002103	bac	g_unclassified_f__Gemmatimonadaceae	Gemmatimonadetes	3
fungi_ASV_8070	0.001831	fungi	g_unclassified_d__Fungi	p_unclassified_d__Fungi	3
bac_ASV_57233	0.001511	bac	Arenimonas	Proteobacteria	3
fungi_ASV_3551	0.001366	fungi	g_unclassified_c__Sordariomycetes	Ascomycota	3
bac_ASV_111908	0.001358	bac	g_unclassified_f__Gemmatimonadaceae	Gemmatimonadetes	3
bac_ASV_1912	0.001345	bac	Subgroup_6	Acidobacteria	3
bac_ASV_72243	0.001286	bac	Marmoricola	Actinobacteria	3
bac_ASV_23271	0.001277	bac	g_unclassified_f__Chitinophagaceae	Bacteroidetes	3
bac_ASV_14856	0.001249	bac	RB41	Acidobacteria	3
bac_ASV_94078	0.001121	bac	Gaiella	Actinobacteria	3
bac_ASV_88010	0.001079	bac	Gaiella	Actinobacteria	3
bac_ASV_78313	0.001059	bac	g_unclassified_o__Gaiellales	Actinobacteria	3
bac_ASV_43006	0.001035	bac	g_unclassified_f__Gemmatimonadaceae	Gemmatimonadetes	3
bac_ASV_68986	0.001031	bac	bacteriap25	Proteobacteria	3
bac_ASV_107859	0.001023	bac	Subgroup_6	Acidobacteria	3
bac_ASV_1554	0.000988	bac	Gemmatimonas	Gemmatimonadetes	3
bac_ASV_32632	0.000977	bac	g_unclassified_f__Gemmatimonadaceae	Gemmatimonadetes	3
bac_ASV_88527	0.000936	bac	g_unclassified_o__Microtrichales	Actinobacteria	3
bac_ASV_106117	0.000867	bac	Pseudolabrys	Proteobacteria	3
bac_ASV_48365	0.000848	bac	Micromonospora	Actinobacteria	3
bac_ASV_74939	0.000843	bac	MB_A2_108	Actinobacteria	3
**fungi_ASV_9957**	0.000824	fungi	Fusarium	Ascomycota	3
bac_ASV_3858	0.000823	bac	UTBCD1	Bacteroidetes	3
bac_ASV_116212	0.000774	bac	g_unclassified_f__Rhodanobacteraceae	Proteobacteria	3
bac_ASV_57923	0.000766	bac	g_unclassified_o__Gaiellales	Actinobacteria	3
bac_ASV_1407	0.000762	bac	Solirubrobacter	Actinobacteria	3

bac_ASV_97047	0.000744	bac	Gemmatimonas	Gemmatimonadetes	3
bac_ASV_83255	0.000702	bac	OLB14	Chloroflexi	3
bac_ASV_5854	0.000699	bac	g_unclassified_f__Gemmatimonadaceae	Gemmatimonadetes	3
**fungi_ASV_2597**	0.000672	fungi	Penicillium	Ascomycota	3
bac_ASV_98389	0.00067	bac	Subgroup_6	Acidobacteria	3
bac_ASV_109318	0.000637	bac	Gaiella	Actinobacteria	3
bac_ASV_32505	0.000635	bac	Subgroup_22	Acidobacteria	3
bac_ASV_110408	0.000616	bac	JG30_KF_CM66	Chloroflexi	3
bac_ASV_40491	0.000612	bac	Nitrospira	Nitrospirae	3
bac_ASV_105696	0.000598	bac	g_unclassified_f__Xanthobacteraceae	Proteobacteria	3
bac_ASV_83795	0.000595	bac	Subgroup_6	Acidobacteria	3
bac_ASV_92898	0.000583	bac	OLB14	Chloroflexi	3
bac_ASV_116223	0.000569	bac	MB_A2_108	Actinobacteria	3
bac_ASV_78343	0.000567	bac	67_14	Actinobacteria	3
bac_ASV_5738	0.000565	bac	g_unclassified_f__Roseiflexaceae	Chloroflexi	3
bac_ASV_88935	0.000562	bac	Adhaeribacter	Bacteroidetes	3
bac_ASV_38771	0.000548	bac	g_unclassified_f__Chitinophagaceae	Bacteroidetes	3
bac_ASV_11052	0.000529	bac	C0119	Chloroflexi	3
bac_ASV_90790	0.000523	bac	SC_I_84	Proteobacteria	3
bac_ASV_33724	0.000499	bac	SC_I_84	Proteobacteria	3
bac_ASV_64907	0.000496	bac	g_unclassified_f__Gemmatimonadaceae	Gemmatimonadetes	3
fungi_ASV_1222	0.000494	fungi	Aspergillus	Ascomycota	3
bac_ASV_115211	0.000485	bac	Rokubacteriales	Rokubacteria	3
bac_ASV_100968	0.000483	bac	67_14	Actinobacteria	3
bac_ASV_85884	0.000479	bac	67_14	Actinobacteria	3
bac_ASV_69862	0.000434	bac	KD4_96	Chloroflexi	3
bac_ASV_20476	0.000419	bac	67_14	Actinobacteria	3
bac_ASV_376	0.000411	bac	Solirubrobacter	Actinobacteria	3
bac_ASV_39351	0.00041	bac	Solirubrobacter	Actinobacteria	3
bac_ASV_48297	0.000404	bac	Bacillus	Firmicutes	3
bac_ASV_116333	0.000403	bac	Phenylobacterium	Proteobacteria	3
bac_ASV_21358	0.00039	bac	Nocardioides	Actinobacteria	3
bac_ASV_84332	0.000384	bac	g_unclassified_f__Chitinophagaceae	Bacteroidetes	3
bac_ASV_10918	0.000375	bac	g_unclassified_f__Roseiflexaceae	Chloroflexi	3
bac_ASV_15298	0.000373	bac	JG30_KF_CM66	Chloroflexi	3
bac_ASV_41412	0.000365	bac	Gemmatimonas	Gemmatimonadetes	3
bac_ASV_25039	0.000363	bac	67_14	Actinobacteria	3
bac_ASV_99637	0.000348	bac	Nordella	Proteobacteria	3
bac_ASV_104902	0.000341	bac	g_unclassified_o__Gaiellales	Actinobacteria	3
bac_ASV_75976	0.000318	bac	g_unclassified_o__Microtrichales	Actinobacteria	3
bac_ASV_25222	0.000315	bac	g_unclassified_f__Steroidobacteraceae	Proteobacteria	3
bac_ASV_59581	0.000315	bac	67_14	Actinobacteria	3
bac_ASV_108889	0.000314	bac	g_unclassified_f__Roseiflexaceae	Chloroflexi	3
bac_ASV_70201	0.000298	bac	Streptomyces	Actinobacteria	3
bac_ASV_116612	0.000289	bac	TRA3_20	Proteobacteria	3
fungi_ASV_9364	0.000288	fungi	Cystofilobasidium	Basidiomycota	3
bac_ASV_74668	0.00028	bac	Nitrospira	Nitrospirae	3
bac_ASV_48494	0.000274	bac	JG30_KF_CM66	Chloroflexi	3
bac_ASV_9345	0.000272	bac	g_unclassified_c__Thermoleophilia	Actinobacteria	3

bac_ASV_13783	0.000261	bac	g_unclassified_f__Intrasporangiaceae	Actinobacteria	3
bac_ASV_27652	0.000254	bac	RBG_13_54_9	Chloroflexi	3
bac_ASV_109335	0.000253	bac	g_unclassified_o__Gaiellales	Actinobacteria	3
bac_ASV_27972	0.000245	bac	g_unclassified_f__Roseiflexaceae	Chloroflexi	3
bac_ASV_57822	0.000222	bac	g_unclassified_o__Actinomarinales	Actinobacteria	3
bac_ASV_14908	0.000217	bac	Rokubacteriales	Rokubacteria	3
bac_ASV_77254	0.000199	bac	Subgroup_6	Acidobacteria	3
bac_ASV_93735	0.000185	bac	Subgroup_6	Acidobacteria	3
bac_ASV_105910	0.000167	bac	Gaiella	Actinobacteria	3
bac_ASV_82788	0.000143	bac	OPB56	Bacteroidetes	3
bac_ASV_40789	0.000099	bac	g_unclassified_f__Gemmatimonadaceae	Gemmatimonadetes	3
bac_ASV_37566	0.000095	bac	Ramlibacter	Proteobacteria	3

Note: The relative abundance of ASVs in modules is arranged in descending order.

Table S4 Functional prediction information of fungal nodes and other negative correlation nodes in main ecological modules

Source	Module	Genus	Phylum	Trophic Mode	Guild	Target	Module	Genus	Phylum	Function_type	Function_group
ASV_4550	2	Fusarium	Ascomycota	Pathotroph–saprotroph–symbiotroph	Animal Pathogen-Endophyte-Lichen Parasite-Plant Pathogen-Soil Saprotroph-Wood Saprotroph	ASV_39013	0	Lysobacter	Proteobacteria	chitinolysis	C-cycle
ASV_5732	2	Plectosphaerella	Ascomycota	Pathotroph	Plant Pathogen	ASV_55972	3	MND1	Proteobacteria	anaerobic_chemoheterotrophy	Energy source
										aerobic_ammonia_oxidation	N-cycle
										nitrification	N-cycle
ASV_2220	3	Talaromyces	Ascomycota	Saprotroph	Undefined Saprotroph	ASV_57233	3	Arenimonas	Proteobacteria	aerobic_chemoheterotrophy	Energy source
ASV_2220	3	Talaromyces	Ascomycota			ASV_88935	3	Adhaeribacter	Bacteroidetes	aerobic_chemoheterotrophy	Energy source
ASV_9957	3	Fusarium	Ascomycota	Pathotroph–saprotroph–symbiotroph	Animal Pathogen-Endophyte-Lichen Parasite-Plant Pathogen-Soil Saprotroph-Wood Saprotroph	ASV_27891	0	Sphingomonas	Proteobacteria	aerobic_chemoheterotrophy	Energy source
ASV_9957	3	Fusarium	Ascomycota			ASV_80436	0	Lysobacter	Proteobacteria	anaerobic_chemoheterotrophy	Energy source
										chitinolysis	C-cycle
ASV_2597	3	Penicillium	Ascomycota	Saprotroph	Undefined Saprotroph	ASV_57233	3	Arenimonas	Proteobacteria	aerobic_chemoheterotrophy	Energy source
ASV_2597	3	Penicillium	Ascomycota			ASV_85322	4	MM2	Proteobacteria	anaerobic_chemoheterotrophy	Energy source

Table S5 Bacterial and fungal interactions within and between co-occurrence clusters across all study samples

Module	Edge	Positive edge	Negative edge
2	443	346	97
3	824	605	219

	Total	Positive Link	Negative Link
Module 1 to Module 2	244	150	94
Module 1 to Module 3	681	510	171
Module 1 to Module 4	842	568	274
Module 2 to Module 3	63	56	7
Module 2 to Module 4	128	94	34
Module 3 to Module 4	699	465	234
